# The Contribution of Particle Swarm Optimization to Three-Dimensional Slope Stability Analysis

**DOI:** 10.1155/2014/973093

**Published:** 2014-06-01

**Authors:** Roohollah Kalatehjari, Ahmad Safuan A Rashid, Nazri Ali, Mohsen Hajihassani

**Affiliations:** ^1^Department of Geotechnics and Transportation, Universiti Teknologi Malaysia, 81310 Skudai, Johor, Malaysia; ^2^Construction Research Alliance, Universiti Teknologi Malaysia, 81310 Skudai, Johor, Malaysia

## Abstract

Over the last few years, particle swarm optimization (PSO) has been extensively applied in various geotechnical engineering including slope stability analysis. However, this contribution was limited to two-dimensional (2D) slope stability analysis. This paper applied PSO in three-dimensional (3D) slope stability problem to determine the critical slip surface (CSS) of soil slopes. A detailed description of adopted PSO was presented to provide a good basis for more contribution of this technique to the field of 3D slope stability problems. A general rotating ellipsoid shape was introduced as the specific particle for 3D slope stability analysis. A detailed sensitivity analysis was designed and performed to find the optimum values of parameters of PSO. Example problems were used to evaluate the applicability of PSO in determining the CSS of 3D slopes. The first example presented a comparison between the results of PSO and PLAXI-3D finite element software and the second example compared the ability of PSO to determine the CSS of 3D slopes with other optimization methods from the literature. The results demonstrated the efficiency and effectiveness of PSO in determining the CSS of 3D soil slopes.

## 1. Introduction

Slope stability analysis is a major concern in projects related to man-made or natural slopes. Several techniques are applied to analyze the stability state of a slope, of which limit equilibrium method (LEM) is the most popular [1]. This method undertakes the static behavior of the slope at the verge of failure and develops equilibriums of the soil body in static condition. Consequently, no stress-strain relationship is considered and corresponding deformation within the soil body is not studied [2]. As a result, the shape of each potential slip surface which defines the lower boundary of sliding body has to be assumed. A numerical ratio as factor of safety (FOS) is used to determine the critical slip surface (CSS) as the least stable slip surface among all potentials. FOS compares the available shear strength of the soil with the existing shear stress (mobilized shear strength) on the assumed slip surface as follows [3]:

(1) $$\begin{array}{c} \text{FOS}\;=\;\frac{S}{T}, \end{array}$$

where *S* is mobilized shear strength force (kN) and *T* is available shear strength of the soil (kN). The mobilized shear strength force is defined as

(2) $$\begin{array}{c} S=\left(\frac{\left[c^{\prime }+\left(\sigma_{n}-u_{w}\right)\tan\phi^{\prime }\right]}{\text{FOS}}\right), \end{array}$$

where FOS is factor of safety, *S* is mobilized shear strength force (kN), *c*′ is cohesion of the soil in terms of effective stress (kN/m^2^), *ϕ*′ is angle of internal friction of soil in terms of effective stress (kN/m^2^), and *σ*_*n*_ is normal stress on the slip surface (kN/m^2^), and *u*_*w*_ is pore water pressure on the slip surface (kN/m^2^). In general, the following principles are required to analyze the stability of a slope within LEM [2].

1. A kinematically admissible slip surface is assumed to define the mechanism of failure.
2. Two static principles as the assumption of plastic behavior for soil mass and validity of Mohr-coulomb failure criterion are employed to determine the shearing strength along the assumed slip surface.
3. Equation of FOS is developed for the assumed slip surface by dividing the available shear strength at the surface by the required shear resistance to bring the equilibrium into limiting condition.
4. An iterative process is used to find the satisfying value of FOS.
5. By using the steps above, a search technique is employed to find the CSS among all assumed slip surfaces.

Although all the LEMs have mutual principles, they differ in utilizing static equilibrium, assumptions, and simplifications. They can be considered as two-dimensional (2D) and three-dimensional (3D) methods. 2D methods simplify the geometry of slopes by transforming the problem into an assumed 2D form. Consequently, some internal and external forces are simplified or ignored in this process. Such simplifications in 2D methods may result in different outcomes form the results of 3D methods. Although the assumptions of 3D methods are mostly derived from the related 2D basics, some new definitions are only available in 3D methods due to plus one dimension that 3D methods have. Ability to consider 3D shapes of slip surface, asymmetric and complex slopes, sliding direction, and intercolumn forces are some of the privileges of 3D methods. However, 3D methods might consider, simplify, or ignore any of these aspects.

Determining the CSS, despite of utilized 2D or 3D method, needs a massive search among possible slip surface. Searching problem is usually defined as optimization problem in engineering. This problem is framed to find appropriate solution among the candidates by minimizing or maximizing an objective function. If more than one solution exists among candidates of a problem, it turns to global optimization. Global optimization methods try to find the global solution, while avoiding local solutions.

Particle swarm optimization (PSO) was initially introduced by Kennedy and Eberhart [4] as a global optimization technique. PSO simulates the birds flock activities when they randomly search for food in their path. Since PSO has been released, its successful application in various engineering problems has begun. The popularity of PSO is mainly due to its comprehensible performance as well as its simple operation [5]. Many researchers applied PSO to solve their problems in the fields of structural [6–8], environmental [9–11], hydrological [12, 13], and geotechnical [14–16] engineering.

Cheng et al. [17] tried to determine the CSS of seven slopes by using PSO as one of the first applications of PSO in slope stability analysis and came to the conclusion that PSO produces appropriate and reasonable results. Furthermore, in a comparison with pattern method, they [17] reported that PSO is capable of finding the global minimum FOS and its related CSS in different slopes. Ever since, PSO has been used progressively as an effective technique to deal with the problem of determining the CSS, to name a few, Cheng et al. [17], Cheng et al. [18], Zhao et al. [19], Tian et al. [20], Li et al. [21], Kalatehjari et al. [22, 23], and W. Chen and P. Chen [24]. However, the contribution of PSO was limited to 2D slope stability problem. In fact, only a few researchers published their results in determining the CSS in 3D slope stability problems and none of them applied PSO [25–30].

Based on the successful performance of PSO in 2D slope stability analysis as well as other problems of geotechnical engineering, it is believed that it can contribute well to determining the CSS of 3D slopes. This paper applies PSO in 3D slope stability problem to determine the CSS of soil slopes. A detailed description of adopted PSO is presented to provide a good basis for more contribution of this technique to the field of 3D slope stability problems.

## 2. Overview of Particle Swarm Optimization 

Kennedy and Eberhart [4] initialized PSO by simulating the behavior of a birds swarm with defined instructions for individual behaviors as well as intercommunications. These instructions help in decision making process of individuals which is based on the following items [4]:

- experience of individual as its best results so far;
- outlay of experience of swarm as the best result among all individuals.

Swarm intelligence as the ability of each individual to use the experience of others guides the swarm toward its optimum goal. Three principals of the swarm behavior in PSO were similar to what described by Reynolds [31].

- Individuals are collision-proof.
- Individuals travel toward swarm objective.
- Individuals travel to the center of swarm.

**Figure 1:**
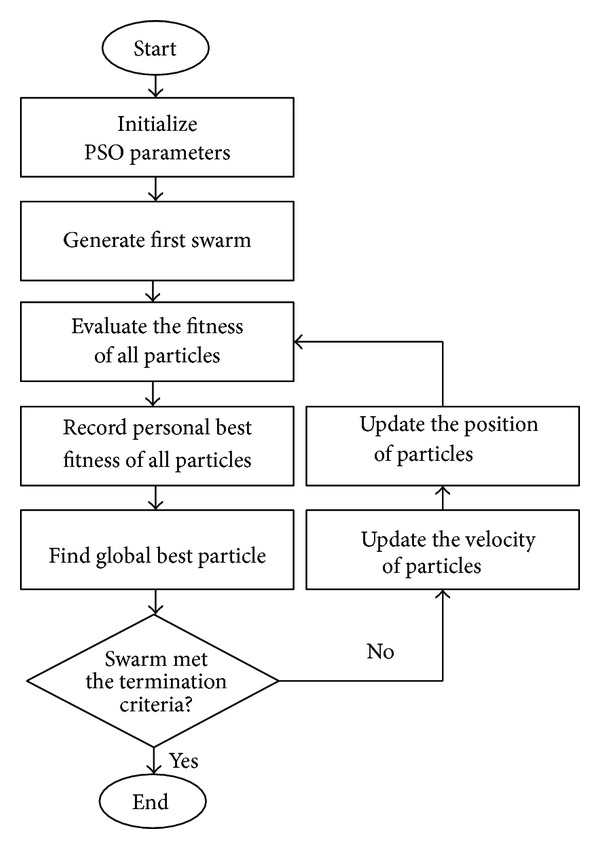
Standard flowchart of PSO.

The standard flowchart of PSO is shown in Figure 1. This process starts by randomly generating a certain number of individuals, namely particles, where each represents a possible solution for the problem [4, 17]. The structure of a particle may contain three sections that separately record its current position, best position so far, and velocity, respectively, as coordinates of current position, coordinates of best position so far, and velocity vectors in a* D*-dimensional space, where* D* starts from one [32]. Consequently, a 3 ×* D*-dimensional particle is fitting for a particle in* D*-dimensional space.

PSO reaches its goal if meets the termination criteria. These criteria are set to guarantee the ending of iterative search process. Appropriate termination criteria are necessary to accomplish a successful search by avoiding premature or late convergence [33]. The commonly used termination criteria are set as follows:

- reaching a maximum number of iterations;
- finding a satisfactory solution;
- achieving a constant fitness for a certain number of iterations.

The closeness of each particle to the best possible solution is defined by the objective function which is aimed to be minimized or maximized by PSO. A fitness function related to the objective function is usually set to calculate fitness value of each particle by assessing its current position. The velocity of particles is determined by (3) based on their best position and global best position in the swarm. To continue the search, (4) updates the position of all particles based on their current position and the obtained velocity. Through an iterative process, the improvement of fitness of particles continues until PSO meets the termination criteria. The global solution is then achieved by the current position of the best particle in the last iteration:

(3) vn(i)=vn(i−1)+u(0,ϑ1)(bpn(i)−xn(i)) +u(0,ϑ2)(bgn(i)−xn(i)),

(4) $$\begin{array}{c} x_{n(i+1)}=x_{n(i)}+v_{n(i)}, \end{array}$$

where *v*_*n*(*i*−1)_ and *v*_*n*(*i*)_ are, respectively, the velocity of *n*th particle in past and current iterations, *u*(0, *ϑ*_1_) and *u*(0, *ϑ*_2_) are the vectors of random numbers of *n*th particle uniformly distributed, respectively, in [0, *ϑ*_1_] and [0, *ϑ*_2_], *bp*_*n*(*i*)_ is the best position of *n*th particle so far, *bg*_*n*(*i*)_ is the position of the best particle of the swarm so far, and *x*_*n*(*i*−1)_ and *x*_*n*(*i*)_ are the positions of *n*th particle, respectively, in the current and the next iterations.

Initial, cognitive, and social parts are three components of velocity equation. The values of *ϑ*_1_ and *ϑ*_2_ in this equation control the exploration and exploitation behaviours of the swarm. While equal values of 2 are commonly used for these parameters in early search, greater values of *ϑ*_1_ and *ϑ*_2_, respectively, provide faster convergence to the solution and enhance discovering the searching space. The velocity of particles may increase surprisingly by adjusting these parameters, so a limiting bound of velocity as [−*v*_max⁡_, *v*_max⁡_] is attached to PSO as constriction coefficients [34]. Shi and Eberhart [35] modified the original equation of velocity to reduce the role of constriction coefficient and introduced (5) by introducing *ω* as the inertia weight of particles. Later on, Clerc and Kennedy [36] demonstrated that inertia weights of greater than one may cause converge problems in PSO and proposed (6) by introducing *ξ* as the constant multiplier in (7). This modification prevents the swarm to explode, guarantees the mature converge, and almost eliminates the need of constriction coefficient. Principally, interior parameters (inertia weight and velocity coefficient) and exterior parameters (swarm size and topology) of PSO should be carefully adjusted to provide the best results:

(5) vn(i)=ωvn(i−1)+u(0,ϑ1)(bpn(i)−xn(i)) +u(0,ϑ2)(bgn(i)−xn(i)),

(6) vn(i)=ξ[vn(i−1)+u(0,ϑ1)(bpn(i)−xn(i))  +  u(0,ϑ2)(bgn(i)−xn(i))],

(7) ξ=2(ϑ1+ϑ1)−2+(ϑ1+ϑ1)2−4(ϑ1+ϑ1),                 (ϑ1+ϑ1)>4.

Topology in the method of intercommunication between particles controls the convergence of a swarm. Topology is divided into static and dynamic categories. In static topologies, the number of connected neighbors to a particle is constant throughout the optimization process. However, this number increases by the progress of optimization process in dynamic topologies to enhance the searching abilities [37].

**Figure 2:**
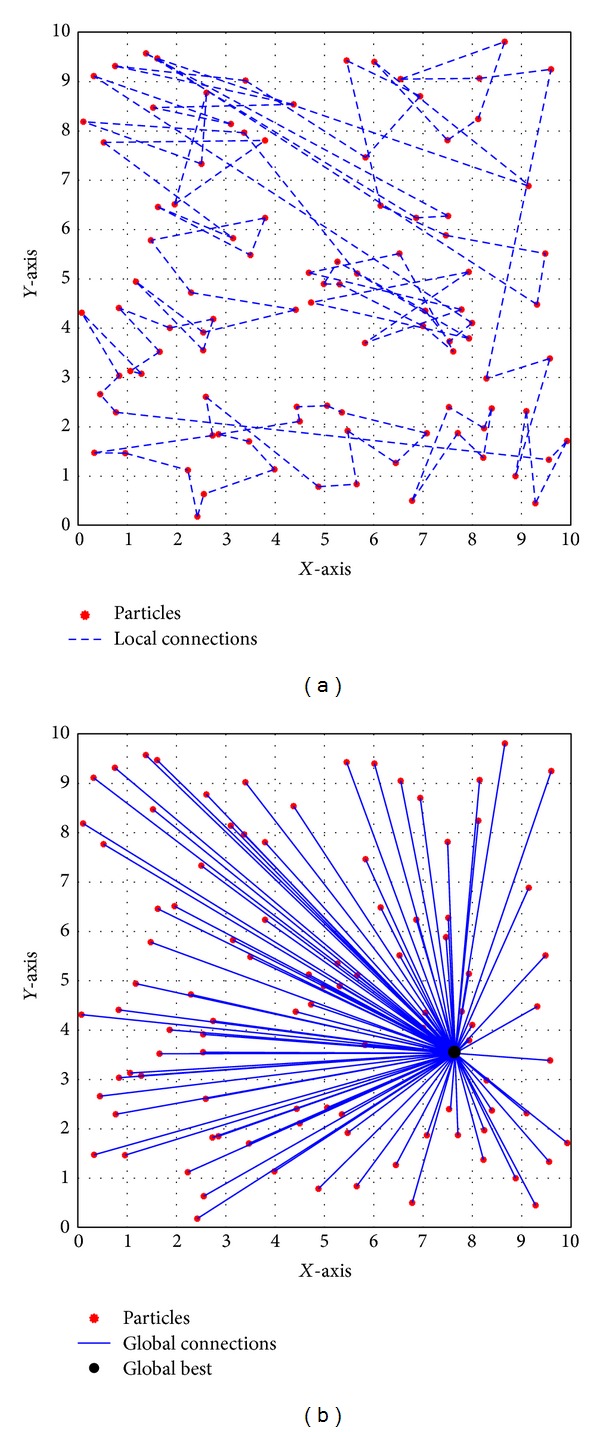
(a) Local and (b) global topologies in 2D search space.

The original PSO aided a conical static topology based on intercommunication of all particles with the global best particle. However, Eberhart and Kennedy [38] proposed another static topology by introducing intercommunication between individuals and local best particles. In this model, each particle was connected to *K* number of its neighbors in the swarm array. The main advantage of this method was the ability of subconvergence in different regions of the search space. Although the convergence of this method was slower than the conical method, it was able to better escape from local optima. For each problem, the appropriate topology can be defined by performing sensitivity analysis on convergence and execution time of PSO.  Figure 2 illustrates conical and local (*K* = 2) topologies for randomly generated 100 particles in a 2D search space.

The size of swarm is defined as the number of its particles. While a small swarm may fail to converge over a global solution, a large swarm may have late convergence. The size of swarm commonly varies from 20 to 50, but the optimum number is usually determined through sensitivity analysis on the convergence parameter of the swarm [36].

## 3. Application of PSO in Slope Stability Analysis

PSO is mainly applied in stability analysis of soil slopes within the framework of LEM [1]. This analysis involves two consequent steps, that is, calculating FOS of candidate slip surfaces and determining the CSS among all candidates [39]. PSO commonly contributes to the second step to determine the shape and location of the CSS which are generally unknown in soil slopes [40].

PSO can be applied in both 2D and 3D slope stability analyses [32]. In 2D analysis, it can be employed to determine the shape of the CSS in a predefined 2D section of the slope. Different shapes are possible for slip surfaces in 2D analysis, such as circular, ellipse, spiral, and polygonal or arbitrary surfaces [22, 41, 42]. In contrast, 3D slip surfaces such as spherical, ellipsoidal, and Nonuniform Rational B-Splines (NURBS) are commonly assumed in 3D analysis [23, 43, 43, 44].

**Figure 3:**
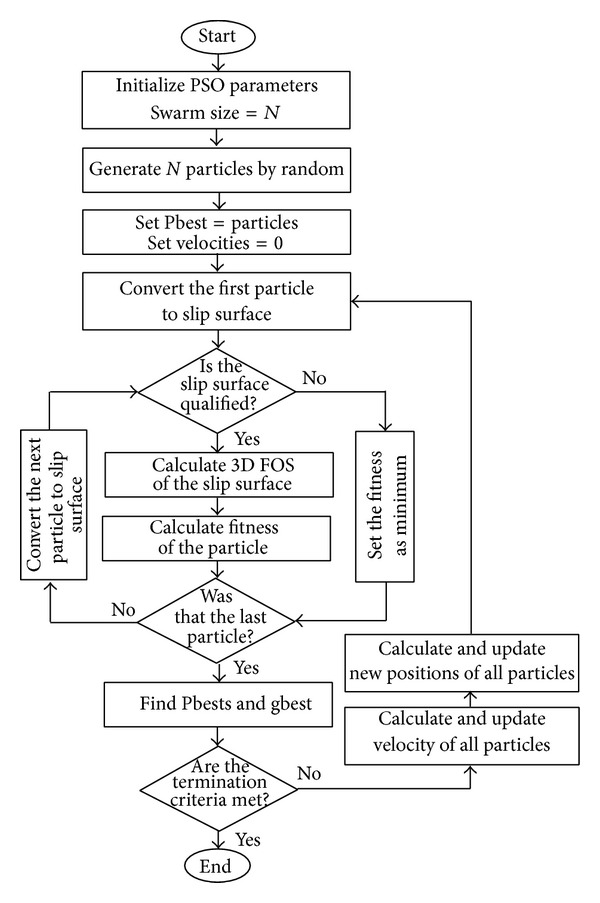
Flowchart of PSO to determine the CSS in slope stability analysis.

Figure 3 shows flowchart of PSO to determine the CSS in slope stability analysis. The optimization procedure is started by setting initial parameters of PSO. Then, a certain number of particles (*N*) is generated in a random pattern over the search space. Since the improvement of swarm has just begun, personal bests of all particles in initial swarm are identical to the particles themselves. Based on the same reason, the velocity of all initial particles is set to zero. After setting up the initial values, the first particle is arranged as its corresponding slip surface. This surface is qualified if it can satisfy the conditions of the problem. Otherwise, it is disqualified. A predefined minimum fitness value is given to disqualify slip surfaces. This value represents a predefined maximum FOS. For a qualified surface, FOS is calculated and the corresponding fitness values of particle are assigned by the fitness function of PSO. This process is repeated for all particles of the swarm.

Current positions of particles that improved their fitness values are recorded to update their personal bests, while previous personal bests are used for other particles. The global best particle is defined by the greatest fitness value in the current swarm. Through an iterative process, subsequent swarms are generated by updating velocities and positions of former particles. The optimization process is terminated by meeting the termination criteria. Eventually, the global best particle of the last swarm represents the CSS of the slope.

### 3.1. Coding of the Particles

**Figure 4:**
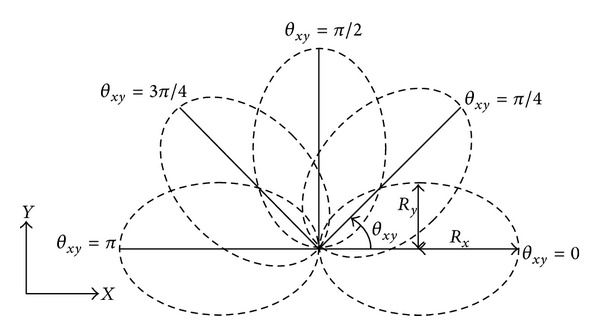
Projection of rotating ellipsoid on *x*-*y* plane.

The structure of particles followed the standard PSO particles involving three sections as current position, previous best position, and the velocity. A rotating ellipsoid was selected as the general 3D shape of slip surfaces. This ellipsoid can rotate on *x*-*y* plane (0 ≤ *θ*_*xy*_ ≤ *π*) to provide various slip surfaces (Figure 4).

In order to achieve the equation of rotated ellipsoid, the parametric equation of general ellipsoid was transferred into new axes by the rotation angle, *θ*_*xy*_. The result presents the rotated ellipsoid in (8). It should be noted that this ellipsoid can be easily transformed to spherical and cylindrical slip surface by, respectively, setting equal three and two semiradiuses:

(8) (cos⁡⁡θxy(x−Xc)−sin⁡θxy(y−Yc))2Rx2  −(cos⁡⁡θxy(y−Yc)+sin⁡θxy(x−Xc))2Ry2  +(z−Zc)2Rz2=1,

where *θ*_*xy*_ is rotation angle of the ellipsoid in *x*-*y* plane, *X*_*c*_, *Y*_*c*_, and *Z*_*c*_ are coordinates of center of ellipsoid in *x*-, *y*-, and *z*-directions, *R*_*x*_, *R*_*y*_, and *R*_*z*_ are semiradiuses of ellipsoid in *x*-, *y*-, and *z*-directions, and *x*, *y*, and *z* are coordinates of an arbitrary point on the surface of ellipsoid.

**Figure 5:**
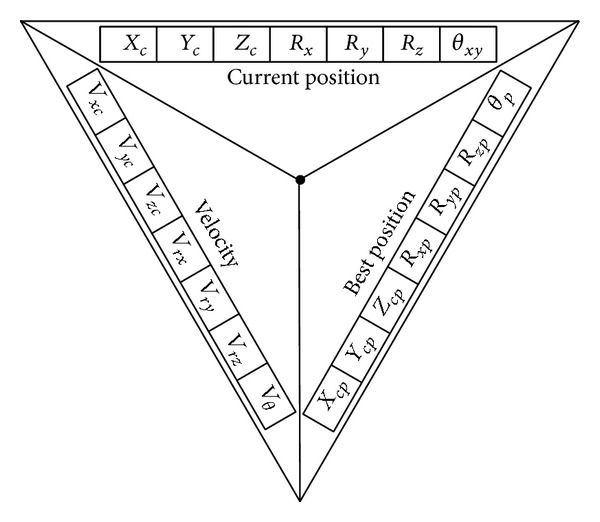
Schematic structures of PSO particles.

Based on the parameters of the rotating ellipsoid, Figure 5 shows schematic structure of a PSO particle. The section of current position records the coordinates of center of ellipsoid, its semiradiuses, and its rotation angle. Considering best position and velocity sections, PSO has seven-dimensional search space and twenty-one-cell particles.

### 3.2. Fitness Function

The quality of particles can be calculated by the fitness function. This function is related to the objective function and provides quantitative tracking of improvement of particles. Consequently, it makes it possible to compare and rank particles in the swarm, where maximum fitness shows the best particle and minimum fitness identifies the worst particle of the swarm. PSO attempts to increase the maximum fitness of swarms during its iterations. Since the objective function of 3D slope stability analysis is equation of FOS, PSO attempts to find the CSS with the minimum FOS by maximizing the fitness value in

(9) $$\begin{array}{c} {\text{Fitness}}_{n(i)}=\frac{1}{\text{FOS}\left(x_{n(i)}\right)}, \end{array}$$

where Fitness_*n*(*i*)_ is fitness value of the *n*th particle in *i*th iteration and FOS(*x*_*n*(*i*)_) is FOS of 3D slip surface described by *x*_*n*(*i*)_.

### 3.3. Sensitivity Analysis on PSO Parameters

**Figure 6:**
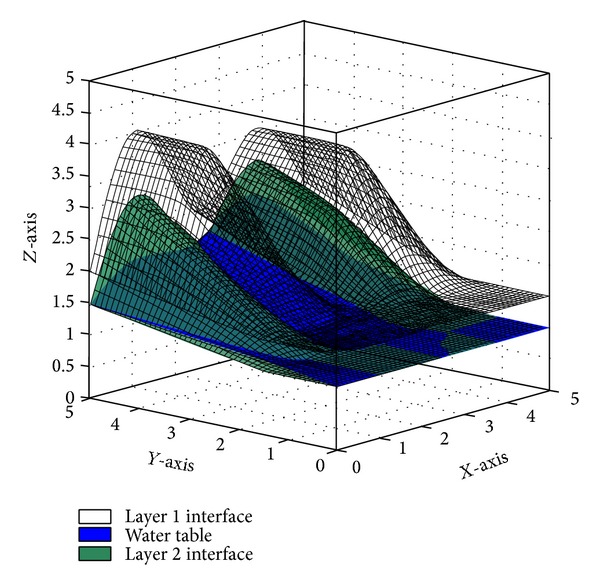
Generated slope model for sensitivity analysis.

The best performance of PSO is guaranteed by initializing appropriate parameters for it. A sensitivity analysis can help to do so. The optimum values PSO parameters in 3D slope stability analysis were defined by designing and performing several independent tests on swarm size, coefficients of velocity, and inertia weight of the swarm. In addition, the convergence behavior of PSO as the average fitness of swarms was observed during the tests. A 3D soil slope was designed with complex geometry, layers of soil, and piezometric line (Figure 6). It should be noted that coding of the study was done by the authors in MATLAB software (Licensed by Universiti Teknologi Malaysia). The overall shape of the slope shows two imbalanced hills with steep sides makes it difficult to find the CSS for conventional slope stability analyses. This specific shape was selected to verify the effectiveness of PSO in complex 3D slopes. The properties of soil layers are described in Table 1.

The size of the swarm is defined based on the condition of search space, dimension of particles, and/or other specifications of the problem. The most common population sizes are 20 to 50 [25]. However, Clerc and Kennedy [36] proposed a relationship to determine the optimum value of swarm size as follows:

(10) $$\begin{array}{c} N_{s}=10+\left[\sqrt{D_{s}}\right], \end{array}$$

where *N*_*s*_ is swarm size, *D*_*s*_ is dimension of the particles, and [ ] is calculator of integer part. Since the dimension of particles in the present problem is seven, the proposed optimum swarm size by this equation is 12. Considering the most common range of the swarm size and the result of equation, an interval of swarm sizes was prepared for sensitivity test. It should be noted that the first swarm of all tests was produced by the same random pattern and the maximum iteration number was set to 100 for all tests.  Table 2 shows the results of tests. The phrase “CPU time” in this table means the exact amount of time that CPU spent on each test. CPU time was used to produce fair comparisons, since some factors including the operating system and available memory can affect the overall duration of the tests.

**Figure 7:**
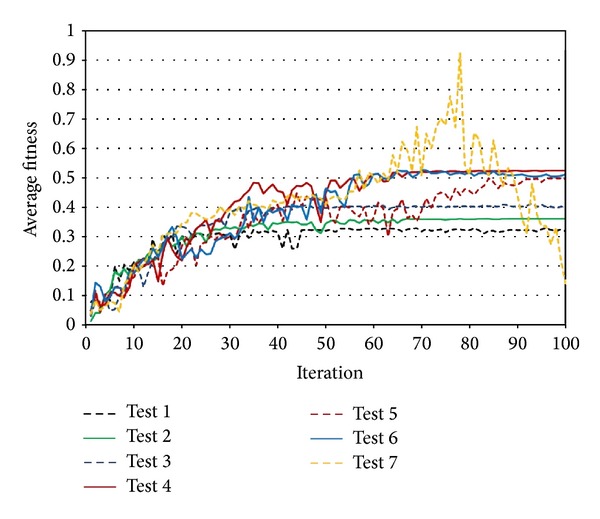
Convergence behavior of PSO with different swarm sizes.

Figure 7 illustrates the convergence behavior of PSO in corresponding swarm sizes of Table 2. Three different convergence behaviors as good, late, and failure can describe these trends. Swarm sizes 5, 15, 25, 35, and 55 provided good convergence over maximum iterations, while swarm sizes 45 and 65, respectively, delivered delay and failure in convergence. Among all the tests, the best convergence was obtained by swarm size of 35 that provided the best convergence with the highest average fitness.

The next tests were performed to find the optimum values of coefficients *ϑ*_1_ and *ϑ*_2_ of velocity equation. Based on the original coefficients of Kennedy and Eberhart [4] and the modified coefficients of Clerc and Kennedy [36], a series of combinations were established as shown in Table 3. All tests were performed by the same initial swarm with the size of 35 (previously obtained as optimum) and the maximum iterations of 100.

**Figure 8:**
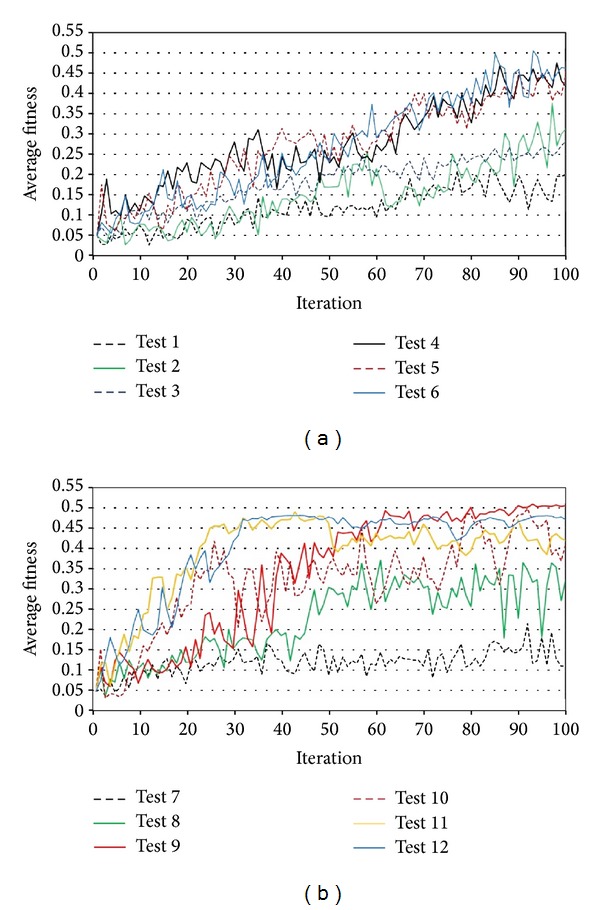
Results of sensitivity tests on (a) unequal and (b) equal coefficients.

The results can be presented in two separated groups including unequal and equal coefficients.  Figure 8 illustrate the results of the tests. The first group failed to converge over the maximum iteration period, but the second group showed different performances. Overall, the best convergence and the greatest average fitness belonged to equal coefficients of 1.75 that makes it the optimum coefficient of velocity equation.

**Figure 9:**
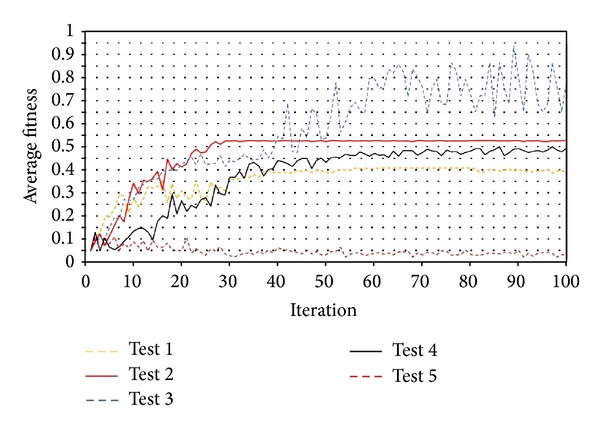
Results of sensitivity tests on inertia weight.

The last sensitivity tests were performed to find the optimum inertia weight (*ω*) of velocity equation. The same initial swarm with size of 35 and equal coefficients of velocity equation as 1.75 (previously defined as optimum values) were applied for all the tests. Five tests with inertia weights of 0, 0.25, 0.5, 0.75, and 1, respectively, were performed based on the proposed values of Shi and Eberhart [35] and Clerc and Kennedy [36].  Figure 9 shows the convergence behavior of PSO in the tests. The results showed successful convergence in all tests, except test 3. It should be noted that test 5 was identical with the original PSO, where no inertia weight was present in velocity equation. Immature convergence has occurred for tests 1 and 2. Although fast convergence appears an advantage at first, it is a sign of trapping a swarm in local solutions. Test 3 failed to converge, test 4 had instable convergence, and test 5 failed to improve its average fitness over the maximum iterations. Consequently, it was not possible to introduce an optimum inertia weight to guarantee the convergence of PSO and improvement of average fitness over the maximum iteration number simultaneously.

**Figure 10:**
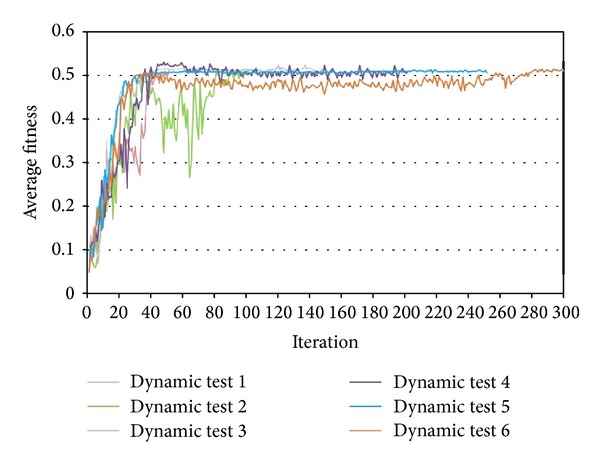
Results of sensitivity tests on dynamic inertia weight.

A dynamic inertia weight was utilized by the present study to overcome the convergence problem of PSO. The proposed strategy started with the most anticonvergence inertia weight (0.5), continued with the normal convergence inertia weight (0.75), and ended up with the most stable convergence (0.25). The switching levels of inertia weights were defined as one-third and two-thirds of maximum iterations.  Figure 10 shows the results of sensitivity tests on dynamic inertia weight with different maximum iterations from 50 to 300 by steps of 50. All tests performed well to converge and improve the average fitness over their maximum iterations, so dynamic inertia weight was adopted for PSO.

## 4. Example Problems

**Figure 11:**
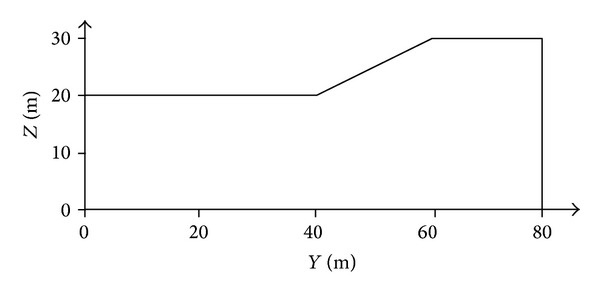
Geometry of 2D section of example problem 1.

**Figure 12:**
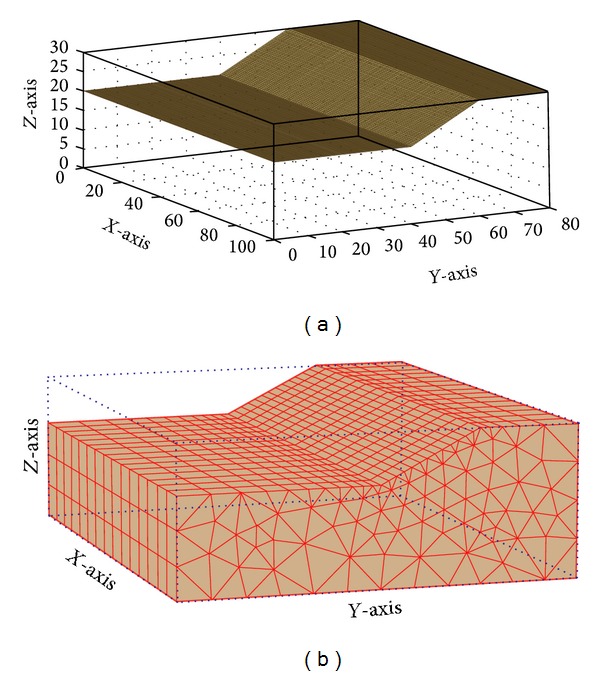
Generated 3D models of example problem 1 by (a) the present study and (b) PLAXIS-3D.

Two example problems were analyzed to verify the performance of PSO in determining the CSS. The properties of the slope materials are shown in Table 4. Example problem 1 was performed to verify the performance of PSO in determining the CSS in comparison with PLAXIS-3D finite element software (License by Universiti Teknologi Malaysia). Alkasawneh et al. [44] applied different search techniques to determine the CSS in 2D slope stability analysis.  Figure 11 illustrates the geometry of the slope. A 3D model was developed based on this 2D section in which the third dimension was extended by 100 meters.  Figure 12 shows the generated 3D models of the slope by the present study and PLAXIS-3D. In both methods, cylindrical slip surface was employed to determine the CSS of the slope.

**Figure 13:**
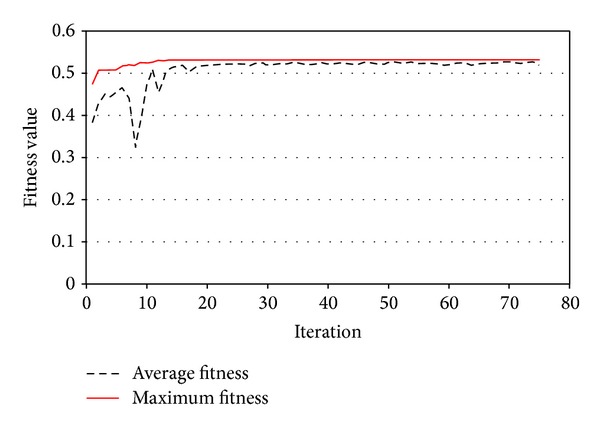
Fitness values versus iterations in example problem 1.

**Figure 14:**
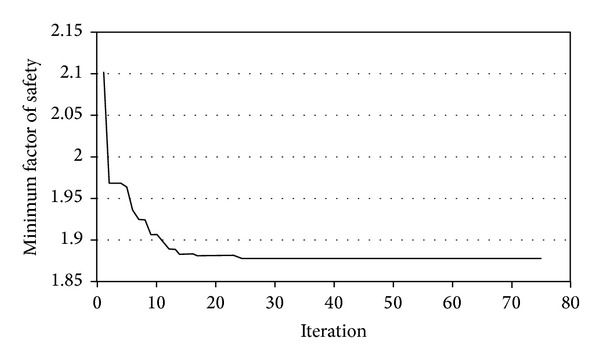
Minimum FOS versus iterations in example problem 1.

**Figure 15:**
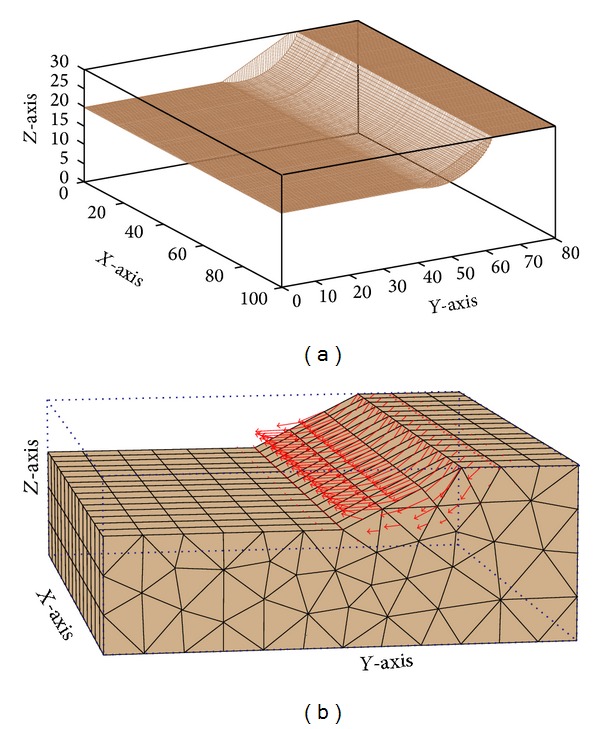
(a) The CSS obtained by the present study and (b) exaggerated displacement vectors of PLAXIS-3D in example problem 1.

PSO improved average and best fitness of the swarm as shown in Figure 13.  Figure 14 shows the minimum FOS versus iterations. PSO provided continuous reduction of FOS to find the CSS. The present study obtained FOS of 1.78 versus the minimum FOS of 1.77 of the PLAXIS-3D.  Figure 15 shows the CSS obtained by the present study and the result of PLAXIS-3D. The present study and PLAXIS-3D obtained similar FOS for the CSS with a small difference of 0.3%. This result demonstrates the ability of PSO to determine the CSS with the minimum FOS in 3D slope stability analysis.

**Figure 16:**
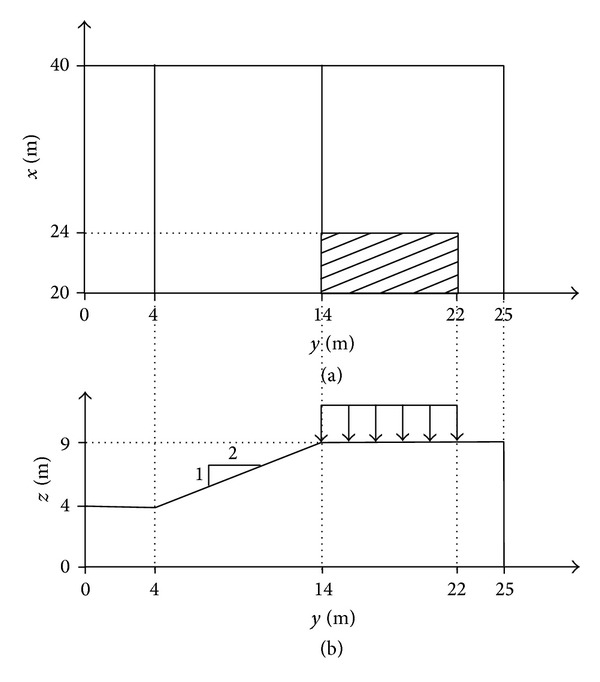
(a) Half-plan view and (b) central cross-section of slope in example problem 2.

Example problem 2 was performed to verify the ability of PSO to determine the CSS with general ellipsoid shape in a comparison with previous studies from the literature. This example was initially analyzed by Yamagami et al. [45] and was reanalyzed by Yamagami and Jiang [25] Yamagami et al. [45] used random generation of surfaces to determine the CSS of this slope, while Yamagami and Jiang [25] employed a combination of dynamic programming and random number generation to do so. It should be noted that the same equation of FOS as previous studies was used to make fair comparison of the results. The example involved a homogeneous slope with gradient of 2 : 1 subjected to a square load of 50 kPa on the top. The uniform load was applied on a square surface with 8 meters sides at the top center of the slope.  Figure 16 illustrates the geometry of example problem 1.

**Figure 17:**
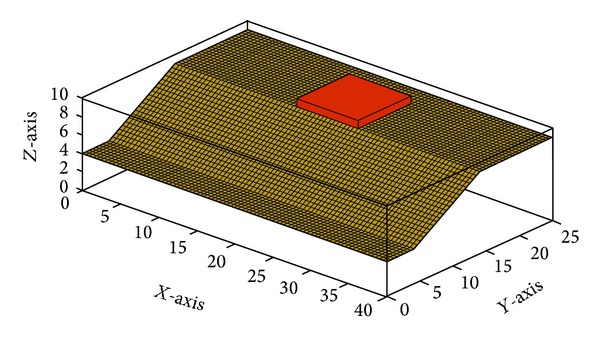
Generated 3D models of example problem 2 by the present study.

**Figure 18:**
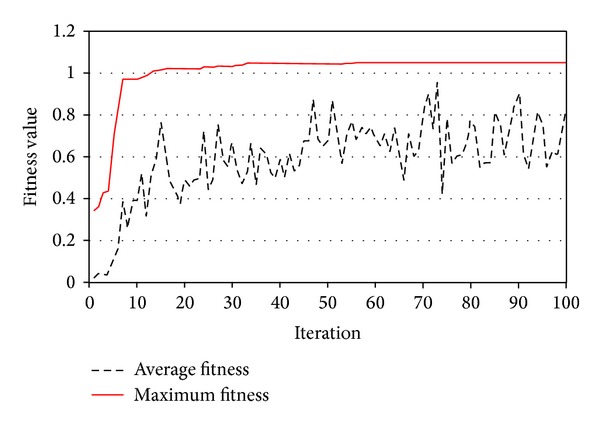
Fitness values versus iterations in example problem 2.

**Figure 19:**
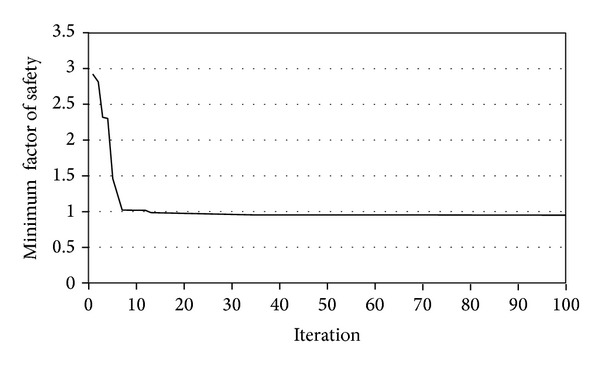
Minimum FOS versus iterations in example problem 2.

Figure 17 shows the generated 3D model by the present study for example problem 2.  Figure 18 plots the process of PSO to improve fitness of the swarm. The trend of average fitness value of the swarm experienced some instability during the process. The main reason of this behavior is the presence of disqualified slip surfaces in the swarm that dramatically decreases the average fitness value. These surfaces were rarely presented in the previous example due to adopted simpler cylindrical shape compared with more complicated ellipsoid shape in this example. The trend of minimum FOS versus iterations is shown in Figure 19. Continuous decrement of FOS by PSO leads to determining the CSS of the slope.

**Figure 20:**
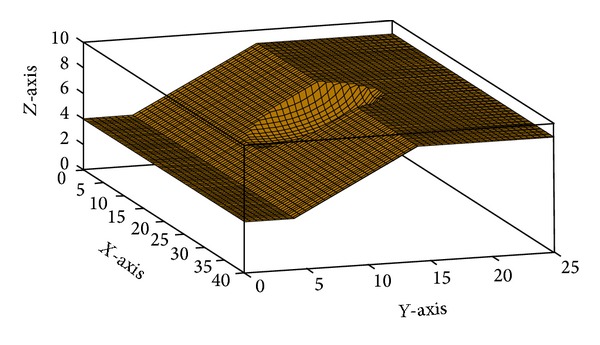
The CSS obtained by the present study in example problem 2.

In spite of similar equation of FOS, the present study found the CSS with a smaller FOS than other methods which is the best result so far. The minimum FOS obtained by PSO was 0.95 compared with 1.14 and 1.03 of random generation of surfaces [45] and DP with RNG [25], respectively. This result demonstrates the ability of PSO to accurately determine the ellipsoid CSS in 3D slope stability analysis.  Figure 20 illustrates the CSS obtained by the present study in example problem 2.

## 5. Conclusion

Determining the critical slip surface of a soil slope is a traditional problem in geotechnical engineering which is still challenging for researchers. This problem needs a massive searching process. Although classical searching methods work for relatively simple problems, they are surrounded by local minima. Moreover, their processes become particularly slow by increasing the number of possible solutions. To eliminate these limitations, PSO has been applied in slope stability analysis based on its successful results in advanced engineering problems. However, this contribution was limited to 2D slope stability analysis. This paper applied PSO in 3D slope stability problem to determine the CSS of soil slopes. A detailed description of adopted PSO was presented to provide a good basis for more contribution of this technique to the field of 3D slope stability problems.

The application of PSO in slope stability analysis was described by presenting a general flowchart. A general rotating ellipsoid shape was introduced as the specific particle for 3D slope stability analysis. In order to find the optimum values of parameters of PSO, a sensitivity analysis was designed and performed. The related codes were prepared by the authors in MATLAB. A 3D model with complex geometry, soil layers, and piezometric line was used in the analysis. This analysis included three steps to find the optimum swarm size, coefficients, and inertia weight of the velocity equation, respectively. Moreover, the performance of PSO to converge over a global optimum solution was verified during the tests. Based on the obtained values of parameters, PSO was prepared for 3D slope stability analysis.

The applicability of PSO in determining the CSS of 3D slopes was evaluated by analyzing two example problems. The first example presented a comparison between the results of PSO and PLAXI-3D finite element software. The second example compared the ability of PSO to determine the CSS of 3D slopes with other optimization methods from the literature. Both of the example problems demonstrated the efficiency and effectiveness of PSO in determining the CSS of 3D soil slopes. Based on the results, it is believed that PSO is highly capable of contributing to the field of 3D slope stability analysis.

## Figures and Tables

**Table 1:** Properties of slope in sensitivity analysis.

	Parameters		
	*γ* (kN/m^3^)	*c*′ (kN/m^2^)	*ϕ*′ (°)
Layer 1	19.78	12	17
Layer 2	17.64	24.5	20

**Table 2:** Results of sensitivity tests on swarm size.

	Test number						
	1	2	3	4	5	6	7
Swarm size	5	15	25	35	45	55	65
Total CPU time (s)	365	625	549	1726	2792	2241	10101

**Table 3:** Combinations of velocity equation coefficients in different tests.

Test number	Relationship	ϑ_1_	ϑ_2_	ϑ_1_ + ϑ_2_
1	ϑ_1_ = 0.25ϑ_2_	0.800	3.200	4
2	ϑ_1_ = 0.50ϑ_2_	1.333	2.667	4
3	ϑ_1_ = 0.75ϑ_2_	1.714	2.286	4
4	ϑ_2_ = 0.25ϑ_1_	3.200	0.800	4
5	ϑ_2_ = 0.50ϑ_1_	2.667	1.333	4
6	ϑ_2_ = 0.75ϑ_1_	2.286	1.714	4
7	ϑ_1_ = ϑ_2_	2.500	2.500	5
8	ϑ_1_ = ϑ_2_	2.000	2.000	4
9	ϑ_1_ = ϑ_2_	1.750	1.750	3.5
10	ϑ_1_ = ϑ_2_	1.500	1.500	3
11	ϑ_1_ = ϑ_2_	1.000	1.000	2
12	ϑ_1_ = ϑ_2_	0.500	0.500	1

**Table 4:** Properties of slopes in example problems.

Properties	*c*′ (kN/m^2^)	*ϕ*′ (degree)	*γ* (kN/m^3^)	*υ*	*E* (kN/m^2^)
Problem 1	15	20	17	0.3	1*E* + 6
Problem 2	10	10	18	—	—
